# The Heuristic Value of *p* in Inductive Statistical Inference

**DOI:** 10.3389/fpsyg.2017.00908

**Published:** 2017-06-09

**Authors:** Joachim I. Krueger, Patrick R. Heck

**Affiliations:** Department of Cognitive, Linguistic, and Psychological Sciences, Brown University, ProvidenceRI, United States

**Keywords:** statistical significance testing, null hypotheses, NHST, Bayes’ theorem, replicability, reverse inference

## Abstract

Many statistical methods yield the probability of the observed data – or data more extreme – under the assumption that a particular hypothesis is true. This probability is commonly known as ‘the’ *p*-value. (Null Hypothesis) Significance Testing ([NH]ST) is the most prominent of these methods. The *p*-value has been subjected to much speculation, analysis, and criticism. We explore how well the *p*-value predicts what researchers presumably seek: the probability of the hypothesis being true given the evidence, and the probability of reproducing significant results. We also explore the effect of sample size on inferential accuracy, bias, and error. In a series of simulation experiments, we find that the *p*-value performs quite well as a heuristic cue in inductive inference, although there are identifiable limits to its usefulness. We conclude that despite its general usefulness, the *p*-value cannot bear the full burden of inductive inference; it is but one of several heuristic cues available to the data analyst. Depending on the inferential challenge at hand, investigators may supplement their reports with effect size estimates, Bayes factors, or other suitable statistics, to communicate what they think the data say.

## Introduction

*The casual view of the p-value as posterior probability of the truth of the null hypothesis is false and not even close to valid under any reasonable model*.

∼Gelman (2013, p. 69)

Gelman’s (2013) observation that many views of *p-*values are too casual to be accurate is itself surprisingly casual. If the *p-*value cannot be equated with the probability of the tested hypothesis, what does it convey? In this article, we explore the association between the *p*-value produced by significance testing and the posterior (after study) probability of the (null) hypothesis. To anticipate our conclusion, we find logical (i.e., built into Bayes’ theorem) and quantitative (after simulation) reasons to think the *p*-value ‘significantly’ predicts the probability of the hypothesis being true. These associations, being neither trivial nor perfect, suggest that the *p*-value is best understood as a useful diagnostic cue for the task of statistical inference. It should neither be ignored nor burdened with the expectation that it reveals everything the researcher wishes to know.

Although our objective is squarely focused on the inductive power of the *p-*value, we find it impossible to dissociate our investigation from the debate over Null Hypothesis Significance Testing. NHST is the preponderant form of significance testing and thus the main producer of *p-*values in psychology and many other fields of empirical research. Yet, the jerry-built framework of NHST invites a host of other types of criticism that lie beyond the scope of this article. For exposition’s sake, we refer to significance testing or specifically to NHST throughout this article as we explore the properties of *p-*values, but this presentational device does not mean that we endorse all aspects of NHST as it is currently practiced.

Significance testing in its various forms has a long tradition in psychological science, and so do statisticians’ concerns and search for alternatives. Significance testing, whether or not it involves null hypotheses, is flawed on logical and probabilistic grounds. It has systematic biases and blind spots. Yet, logical and methodological limitations afflict all methods of inductive inference (García-Pérez, 2016). Hume (1739/1978) famously observed the impossibility of a rational justification of inductive inference. The question he asked, and which we should ask today, is a pragmatic one: how well does a method perform the task placed before it? And by what criteria can we judge a method’s worth? In psychological science, much of the critical debate has been focused on NHST, presumably because many researchers use it ritualistically with a narrow focus on the *p*-value, and without understanding its meaning (Meehl, 1998; Gigerenzer, 2004; see also Mayo, 1996; Perezgonzalez, 2015b). Greenland et al. (2016) list no fewer than 25 misconceptions regarding *p*, chief among them the idea that *p* reflects the probability of the research hypothesis being true, that is, Gelman’s gripe. Here, we can only briefly sketch the main themes of criticism before considering a specific set of questions in greater depth: what is the association between the *p-*value and the revised probability of the tested hypothesis? What are some of the factors that affect this association? Should these factors matter to the working researcher?

We address these questions with computer simulations. As we progress, it will become clear that we freely draw from distinctive statistical traditions, including Fisher’s framework, the Neyman–Pearson paradigm, and Bayesian ideas. We follow this eclectic and pragmatic route in order to obtain answers to our chief questions that may translate into applied practice. We will conclude with reflections on the place of the *p-*value in psychological research and the role it may play in informing, however tentatively, theoretical considerations. Seeing some value in the use of the *p*-value, we do not end with a wholesale condemnation of significance testing (while granting that there may be other sufficient reasons). If, in the course of events, significance testing is abandoned or replaced with, for example, estimation methods (Cumming, 2014) or techniques of Bayesian model comparison (Kruschke, 2013; Kruschke and Lidell, 2017), our analysis might be remembered as a requiem for significance testing and NHST. Then, looking back from the future, we may come to see what we have lost, for better or for worse.

## A Brief History of Criticism

A radical conclusion from the critical reception of significance testing is surgical: remove such testing and the *p*-value from research altogether (e.g., Schmidt and Hunter, 1997). Indeed, the journal *Basic and Applied Social Psychology* no longer accepts research articles reporting significance tests (Trafimow and Marks, 2015), while *Psychological Science* nudges authors toward other “preferred methods” (Eich, 2014).^1^ We think it self-evident that a decision to ban any particular method should clear a rational threshold. Perhaps a ban is justified if significance testing (and the resulting *p*-value) causes more harm than good. Some believe this to be so (Ioannidis, 2005; but see Fiedler, 2017), but harm and good are elastic concepts; they are difficult to define and measure in a probabilistic world. A more cautious position is to say that the *p-*value should be abandoned if its contribution to scientific progress is too small and if other measures perform better. Here, a difficulty lies in what is meant by ‘too small,’ or ‘better.’ Recall Hume’s skepticism regarding the appraisal of induction. Scientists trying to evaluate a particular method have no access to truth outside of the inductive enterprise itself – if they did, they would not need induction. A method of inductive inference can be evaluated only indirectly with the help of other inductions. Recognizing this constraint, we attempt to estimate the usefulness of the *p-*value by pragmatically relying on other (mainly Bayesian) modes of induction.

Criticism of *p*-values and significance testing takes several forms. One prominent concern is that researchers misunderstand the process of inference and fail to comprehend the meaning of the *p*-value (Bakan, 1966; Cohen, 1994; Goodman, 2008; Bakker et al., 2016; Greenland et al., 2016). Gelman’s epigraphic warning is a notable expression of this view. Another, more serious, criticism is that researchers deliberately or unwittingly engage in practices resulting in depressed *p*-values (Simmons et al., 2011; Masicampo and Lalande, 2012; Head et al., 2015; Perezgonzalez, 2015b; Kunert, 2016; Kruschke and Lidell, 2017). For our purposes, it is essential to note that both these criticisms are matters of education and professional ethics, which need to be confronted on their own terms. We will therefore concentrate on criticism directed at the intrinsic properties of *p.* Chief among these is the recognition that *p*-values show a high degree of sampling variation (Murdoch et al., 2008; Cumming, 2014). Variability suggests unreliability, and unreliability limits validity. The strongest reaction is to conclude that the evidentiary value of *p* is highly uncertain, or even nil. By implication, all substantive claims resting on significance testing should be ignored. Again, this may be an over-reaction. We know of no critics willing to ignore the entire archival record built on significance tests. Can we truly say that we have learned nothing (Mayo, 1996)? If we have learned something, the question is: how much?

Assuming that significance testing has taught us *something*, there remains a strong concern that much of what we think we have learned is – or will turn out to be – false (Murayama et al., 2014). Significance testing is not neutral with respect to the hypothesis being tested. At the limit, as samples become very large, even very small deviations from the hypothesized point (e.g., 0) will pass the significance threshold (Kruschke, 2013; Kruschke and Lidell, 2017). Significance testing is thus biased against the hypothesis being tested (Greenwald, 1975; Berger and Sellke, 1987). Even when the statistical hypothesis (most often the null) is true, the *p*-value will be < 0.05 in 5% of the cases, and by definition so (Lindley, 1957; Wagenmakers et al., 2016). At the same time, there is also the concern that most empirical samples are not large enough to detect important effects (Cohen, 1962; Sedlmeier and Gigerenzer, 1989). That is, significance testing is not only liable to produce false positives, but also false negatives. Increases in statistical power – which is typically achieved with increases in sample size – will lower *p*-values (see Hoenig and Helsey, 2001, for a formal proof). Both of these (seemingly opposite) concerns, the risk of false positives and the risk of false negatives, imply that many exact replications will fail (Open Science Collaboration, 2015).^2^ The meta-problem of uncertain (and low) replicability has caught the attention of the scientific community as well as the general public as it goes to the heart of the question of how much of a contribution scientific research can make to the well-being of those who pay for it.

More criticism does not always do more damage. The idea that *p-*values have no validity conflicts with the view that samples are too small. Yet, both lines of criticism raise the specter of false positives results. Anticipating this concern, Fisher (1935/1971) recommended a *p*-value of 0.05 as a prudent threshold the data should pass before meriting the inference of significance. He regarded this threshold as a *heuristic* rather than a firm or logical one and the *p*-value as a “crude surprise index.” “No scientific worker,” Fisher (1956, p. 42) wrote, “has a fixed level of significance at which from year to year, and in all circumstances, he rejects hypotheses; he rather gives his mind to each particular case in the light of his evidence and his ideas.” A variant of the idea that significance testing is biased toward ‘positive’ results is the argument that the method does not allow for a corroboration of the tested hypothesis. It cannot, by design, detect true negatives. There is only refutation but no confirmation. Some Bayesian scholars consider it critical that the evidence must be allowed to support the inference that the tested hypothesis is indeed true (Kruschke and Lidell, 2017; Rouder et al., 2017). According to this view, it is a prime task of scientific research to detect and document ‘invariances,’ that is, to show that important phenomena *do not change* even when salient contextual factors suggest that they would (Wagenmakers, 2007; Rouder et al., 2009).^3^ Conversely however, and as noted above, significance testing may also miss true effects due to lack of power or precision in measurement (Dayton, 1998; Vadillo et al., 2016) and it may thereby retard scientific exploration (Fiedler et al., 2012; Baumeister, 2016).

One general response to these diverse and partially contradictory criticisms is to place one’s hope in very large samples. The call ‘Let the data be big!’ might draw more applause were it not for the ecological constraints of laboratory research and reduced efficiency of scientific work. Baumeister (2016) recalls that 10 observations per cell used to be the standard in social psychology, but that recently expectations have risen fivefold. Baumeister observes that a commitment to gather very many observations will decelerate the trial-and-error exploration of creative ideas. Sakaluk (2016) observes that many researchers must work with small to medium samples because they lack the resources to collect large samples for every scientific question they ask. Classic methods were developed to provide small-sample statistics whose fidelity should be evaluated. Aside from such constraints, the pursuit of large samples is understandable. Large samples make estimates more reliable and reduce error. In a very large sample, the obtained effect size (for example, *d*) approximates the population effect size (δ) and the *p*-value is highly diagnostic. If the null hypothesis is false, *p* converges on 0; if the null is true, the probability of a false positive is 0.05. Any reduction in sample size reduces this validity, but does not eliminate it.^4^ As part of our investigation, we will explore the effect of increasing sample size on the two types of errors, false positives and false negatives.

## The Bayesian Context

If one is to reject a statistical hypothesis, there needs to be sufficient reason for the belief that the hypothesis is false. There needs to be an estimate of the probability of the hypothesis being true given the data, or p(H|D). However, the standard *p*-value is the inverse of this conditional probability, namely the probability of the data (or data more extreme) given the hypothesis, p(D|H) (Wasserstein and Lazar, 2016). When researchers reject the hypothesis, they have presumably inferred a low p(H|D) from a low p(D|H). They cannot simply equate these two conditional probabilities because this would assume a symmetry that is rare in the empirical world (Dawes, 1988; Gelman, 2013). Conversely, they cannot assume that p(D|H) tells them nothing. Kruschke and Lidell (2017) warn that “the frequentist *p-*value has little to say about the probability of parameter values.” But how much is little? A lack of symmetry does not mean a lack of association. If there is a positive association between p(D|H) and p(H|D), the former has heuristic validity for the estimation of the latter.

Bayes’ Theorem formalizes the matter of inverse probability (Jeffreys, 1961; Lindley, 1983). Before turning to the likelihood version of Bayes’ theorem, which is preferred in formal analysis, we consider the probability version, which is more familiar. Here, the probability of the hypothesis given the data is equal to the probability of the data given the hypothesis times the ratio of two unconditional probabilities:

$$\begin{array}{c} p\left(H|D\right)=p\left(D|H\right)\times \frac{p\left(H\right)}{p\left(D\right)} \end{array}$$

The unconditional probability of the hypothesis, p(H), is its prior probability, that is, the estimated probability of this hypothesis being true in the absence of evidence. The unconditional probability of the data, p(D), is the probability of the empirical evidence found in light of *any* hypothesis, which comprises the statistical hypothesis (H) and its alternative(s) (∼H). Bayes’ Theorem can thus be written as:

$$\begin{array}{c} p\left(H|D\right)=\frac{p\left(H\right)\times p\left(D|H\right)}{p\left(H\right)\times p\left(D|H\right)+p\left(\sim H\right)\times p\left(D|\sim H\right)} \end{array}$$

The theorem teaches two lessons. First, to simply equate p(H|D) with p(D|H) is to commit a fallacy of reverse inference (Krueger, 2017). Second, to dismiss p(D|H) is to ignore the fact that it is one of the determinants of p(H|D) (Nickerson, 2000; Krueger, 2001; Trafimow, 2003; Hooper, 2009).

Some scholars have noted the association between the *p-*value and the posterior probability of the hypothesis (Greenland and Poole, 2013). Using simple assumptions (see below), one of us estimated the association between p(D|H) and p(H|D) to be *r* = 0.38 (Krueger, 2001). This result offered a clue for why many researchers continue to use practice of significance testing, but it was too weak to have normative force. Trafimow and Rice (2009) replicated this result and concluded that significance testing has little value. How large should this correlation be? It would be reassuring to see a correlation as large as a typical reliability coefficient, that is, a coefficient greater than 0.70. Reliability coefficients rise with the reduction of measurement error. Yet, the correlation between p(D|H) and p(H|D) is not a matter of reliability but a matter of predictive validity. Even if both probabilities were measured with precision, they would not be perfectly correlated. Beliefs of what constitutes an acceptable level of predictive validity vary. For measures that are considered subtle and sensitive, even validity correlations of around 0.3 have been presented as feats of prediction (e.g., Greenwald et al., 2009). We propose that a validity correlation of 0.5 is large enough to warrant scientific and practical interest. This is a realistic aim, and we ask if the *p*-value can meet it.

## Sampling Probabilities

How well does the *p*-value, p(D|H), predict the criterion measure, p(H|D), that researchers seek when conducting a significance test? Bayes’ Theorem implies a positive association. As the *p*-value falls, so does the criterion of truth, p(H|D). If p(H) and p(D|∼H) were constant, the correlation between p(D|H) and p(H|D) would be perfect. Krueger (2001) and Trafimow and Rice (2009) assumed flat and independent distributions for p(H), p(D|H), and p(D|∼H). We replicated their finding (*r* = 0.372) with 100,000 sets of three input probabilities drawn randomly from uniform distributions. The distribution of p(H) was bounded by 0 and 1 and the distributions of p(D|H) and p(D|∼H) were bounded by 0 and 0.5. We then proceeded to use both likelihood ratios and probabilities to compute p(H|D) and we found very similar results. Here, we report only the results obtained with likelihood ratios in line with the Bayesian notion that “only the data actually observed – and not what might have occurred – are needed, so why use the might-have-been at all? (Lindley, 1983, p. 6).^5^ Compared with probability ratios, likelihood ratios are less biased against the null hypothesis.^6^ When using likelihoods to compute p(H|D), the criterion correlation between p(D|H) and p(H|D) dropped to *r* = 0.263.^7^

**FIGURE 1:**
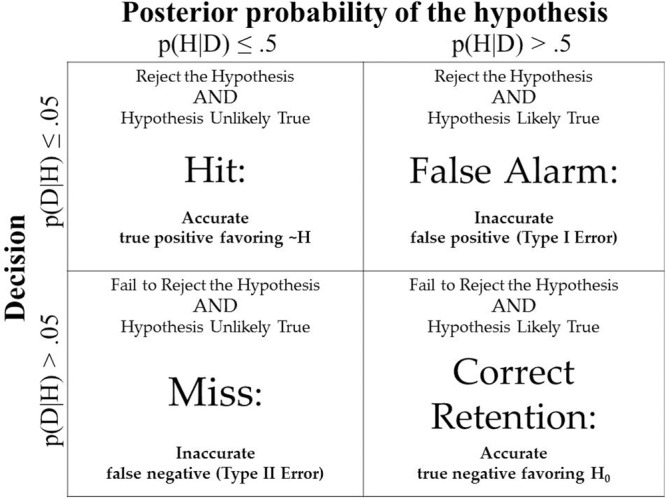
The decision-theoretic context of significance testing.

Assuming that researchers reject a hypothesis when *p* < 0.05, we asked whether the posterior probability was less than 0.5, that is, whether the hypothesis was more likely to be false than true. This threshold is a heuristic choice; it is prudent in that it avoids judgments of value, importance, or need. Other (especially lower) thresholds may be proposed in light of relevant utility considerations (Lindley, 1983). We then categorized each of the 100,000 simulated experiments in a decision-theoretic outcome table (cf. Swets et al., 2000). The rejection of an improbable hypothesis is a Hit in that this hypothesis is less likely than its alternative in light of the data. In contrast, the rejection of a hypothesis that is still more probable than its alternative is a False Alarm. The retention of a probable statistical hypothesis is a Correct Rejection in standard decision-theoretic terms, but we will refer to it as a Correct Retention (i.e., retaining a probable hypothesis) for ease of exposition. Finally, the failure to reject an improbable hypothesis is a Miss. **Figure 1** displays the four decision-theoretic outcomes^8^.

**FIGURE 2:**
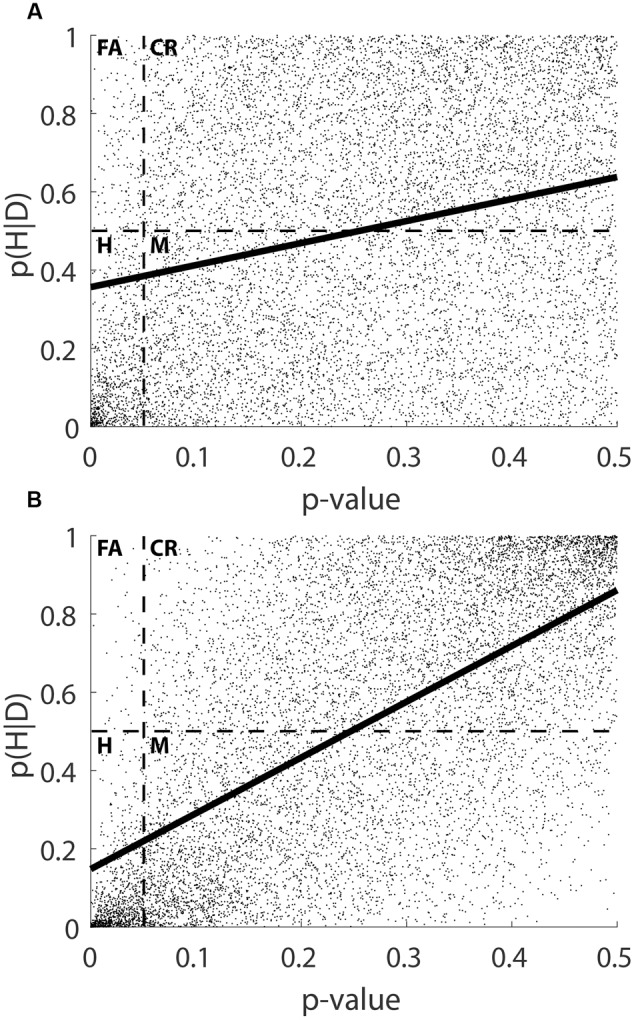
Distribution of p(D|H) and p(H|D) when drawing input terms from uniform distributions. Dashed lines indicate boundary points for classification, with the resulting rectangles capturing each category type (bottom left: Hit; top left: False Alarm; bottom right: Miss; top right: Correct Retention). **(A)** Shows the case for *r*(p(H),p(D|H)) = 0; **(B)** shows the case for *r*(p(H),p(D|H)) = 0.5.

**Figure 2A** plots the posterior probability of the hypothesis, p(H|D), against the *p-*value, p(D|H). A linear model predicts p(H|D) as 0.585p(D|H) + 0.359; *R*^2^ = 0.072. For *p* = 0.05, 0.01, and 0.001, respectively, p(H|D) = 0.389, 0.365, and 0.360. The plot shows a mild concavity, and a second-order polynomial model provides a slightly better fit with -2.352p(D|H)^2^ + 1.735p(D|H) + 0.267; *R*^2^ = 0.092. The predicted values for p(H|D) are 0.348, 0.284, and 0.269 for the three benchmarks of *p*. That is, the predicted posterior probability of the hypothesis is in each case below 0.5. Yet, these predicted posterior probabilities are not as low as the corresponding *p*-values, and they decrease more slowly. Statistical regression guarantees this result.^9^

**Figure 2A** and the top of **Table 1** show the classification of the results. With *p* = 0.05, there are few False Alarms (1.94%). The division of the percent of False Alarms by the total percent of significant results (Hits + False Alarms) yields a ‘false alarm ratio’ (Barnes et al., 2009). We find that for 19.34% of the significant results the null hypothesis remains more probable than its alternative. A ‘miss ratio’ is obtained by dividing the percent of Misses by the total percent of non-significant results (Misses + Correct Retentions, 42.03/[42.03+47.95]). For 46.71% of the non-significant results, the null hypothesis is less probable than its alternative. The middle and the bottom parts of **Table 1** show that as the *p-*value decreases to 0.01 and 0.001, the false alarm ratio decreases, whereas the miss ratio does not change. In other words, setting a more conservative criterion for the rejection of the hypothesis provides better insurance against false positive inferences, although it does not protect against missing important effects.^10^

**Table 1:** *Crossed proportions of conditional probability terms (p < 0.05)*.

	p(H|D) ≤ 0.50	p(H|D) > 0.50
p(D|H) ≤ 0.05	8.080	1.937
p(D|H) > 0.05	42.030	47.953

*Crossed proportions of conditional probability terms (p < 0.01).*		

	**p(H|D) ≤ 0.50**	**p(H|D) > 0.50**

p(D|H) ≤ 0.01	1.89	0.14
p(D|H) > 0.01	48.38	49.59

*Crossed proportions of conditional probability terms (p < 0.001).*		

	**p(H|D) ≤ 0.50**	**p(H|D) > 0.50**

p(D|H) ≤ 0.001	0.22	0.00002
p(D|H) > 0.001	49.40	50.38

Bayes’ Theorem treats prior and conditional probabilities as conditionally independent. For any value of p(H), p(D|H) is – in theory – free to vary. Yet, the assumption of independence may not hold in empirical research. Theoretical considerations, past research, and experience-based hunches allow researchers to gauge the riskiness of their hypotheses (Meehl, 1998; Kruschke, 2013; Kruschke and Lidell, 2017). Doing so, researchers will select hypotheses non-randomly, and as a result, the prior probability of the hypothesis, p(H), and the obtained *p-*values become positively correlated. A risky alternative hypothesis (∼H, e.g., Uri can mentally bend spoons when primed with the name ‘Geller’) means that the probability of the statistical null hypothesis, p(H), is high and it makes a non-significant outcome (p(D|H) > 0.05) likely. With a large effect (∼H: δ = 0.8) being initially either probable (p(H) = 0.1) or improbable (p(H) = 0.9), data will more likely be sampled from the ∼H or the H distribution, respectively. The *p*-value will be smaller in the first case than in the second case, which yields a positive correlation between p(H) and p(D|H). As the effect (d) becomes smaller, the same argument holds, but less strongly so.^11^

We will elaborate this argument in a simulation below. For now we treat it as an ecological constraint and we consider a simulation in which the correlation between p(H) and p(D|H) varied from 0 to 0.9 in steps of 0.1. **Table 2** shows a sharp rise in the criterion correlation between p(D|H) and p(H|D), but only small changes in the prevalence of the two types of error and the overall accuracy of classification (the phi coefficient). Consider the case of *r*(p(H),p(D|H)) = 0.5. The criterion correlation is 0.628 and p(H|D) is predicted as 1.4p(D|H) + 0.159, *R*^2^ = 0.395 (see also **Figure 2B**). For *p* = 0.05, 0.01, and 0.001, respectively, the predicted values of p(H|D) are 0.229, 0.173, and 0.160. The polynomial model is -1.683p(D|H)^2^ + 2.243p(D|H) + 0.088; *R*^2^ = 0.404, with predicted values of p(H|D) being 0.207, 0.111, and 0.090. In short, the *p*-value predicts the posterior probability of the hypothesis more effectively if it is already correlated with the prior probability. As a comparison, we ran a simulation using a negative correlation, *r* = -0.5, between p(H) and p(D|H), and found a criterion correlation of -0.189. These results suggest that the *p*-value works well when it should, and that it does not when it should not.

**Table 2:** Positive correlation between p(H) and p(D|H).

*r*(p(H),p(D|H))	*r*(p(D|H),p(H|D)	FA ratio	Miss ratio	Phi
0	0.267	0.200	0.465	0.201
0.1	0.343	0.157	0.460	0.229
0.2	0.415	0.120	0.449	0.260
0.3	0.494	0.092	0.444	0.278
0.4	0.565	0.063	0.436	0.302
0.5	0.628	0.046	0.430	0.313
0.6	0.698	0.031	0.425	0.327
0.7	0.760	0.018	0.416	0.338
0.8	0.826	0.008	0.411	0.349
0.9	0.891	0.003	0.405	0.356

*FA, false alarm.*

We then asked how the correlation between *p* and the probability of the data under the alternative hypothesis, p(D|∼H) affects posterior probabilities. Strong theory provides clear alternatives to the statistical null hypothesis so that the data are either probable under the null or probable under the alternative. In other words, the correlation between p(D|H) and p(D|∼H) should be negative *a priori*. **Table 3** shows that over a range from 0 to -0.9 for this correlation, the criterion correlation became stronger, the false alarm ratio dropped, and the miss ratio varied little. We also used a positive correlation [*r* between p(D|H) and p(D|∼H) = 0.5] as input and found a very low criterion correlation to *r* = 0.132. In short, a research design that pits two hypotheses against each other so that the data cannot be improbable (or probable) under both allows the *p-*value to reach its greatest inductive potential.

**Table 3:** Negative correlation between p(D|H) and p(D|∼H).

*r*(p(D|H),p(D|∼H))	*r*(p(D|H),p(H|D)	FA ratio	Miss ratio	Phi
0	0.260	0.198	0.468	0.199
–0.1	0.287	0.181	0.464	0.213
–0.2	0.311	0.165	0.462	0.225
–0.3	0.345	0.144	0.462	0.236
–0.4	0.363	0.144	0.463	0.234
–0.5	0.390	0.135	0.461	0.242
–0.6	0.411	0.132	0.461	0.245
–0.7	0.437	0.126	0.459	0.249
–0.8	0.461	0.123	0.463	0.248
–0.9	0.492	0.125	0.456	0.253

To recapitulate, we saw in the first set of simulations that [1] the *p*-value predicts the posterior probability of the tested hypothesis, [2] this correlation is strongest under the most realistic assumptions, [3] false positive inferences are least likely under the most realistic settings, and that [4] the probability of false negative inferences (Misses) is high. The *p*-value thus appears to have heuristic value for inductive inference. Yet, these simulations are only first approximations. They were limited in that input correlations varied only one at a time. Further, these simulations did not involve a sampling of data from which correlations were computed; they instead sampled probability values and stipulated specific correlations among them. We designed the next round of simulations to address these limitations.

## Sampling Observations

To obtain values for p(D|H) and p(D|∼H) from sampled data, we generated sets of two normal distributions with 100,000 cases each. In each set, one distribution (*M* = 50, *SD* = 10) was paired with an alternative distribution (*M* ranging from 50.1 to 60 in steps of 0.1 and *SD* = 10). Standardized effect sizes, δ, thus varied from 0.01 up to 1.0. We then drew mixed samples of 100 observations from each pair of populations, letting the number of observations drawn from the lower distribution range from 10 to 90 in steps of 10. We drew 50 sets of samples for each combined setting of effect size and mixed sampling to generate distributions of means. For each of these 900 distributions, we obtained the *z* score, its one-tailed values of p(D|H) and p(D|∼H), and the corresponding probability densities. Finally, we varied the prior probability of the hypothesis that μ = 50, p(H), from 0.01 to 0.99 in steps of 0.01 for each of these 900 *p*-values. This process yielded a total of 89,100 simulation experiments [100 steps of δ ^∗^ 9 steps of sampling proportions ^∗^ 99 levels of p(H)].

**FIGURE 3:**
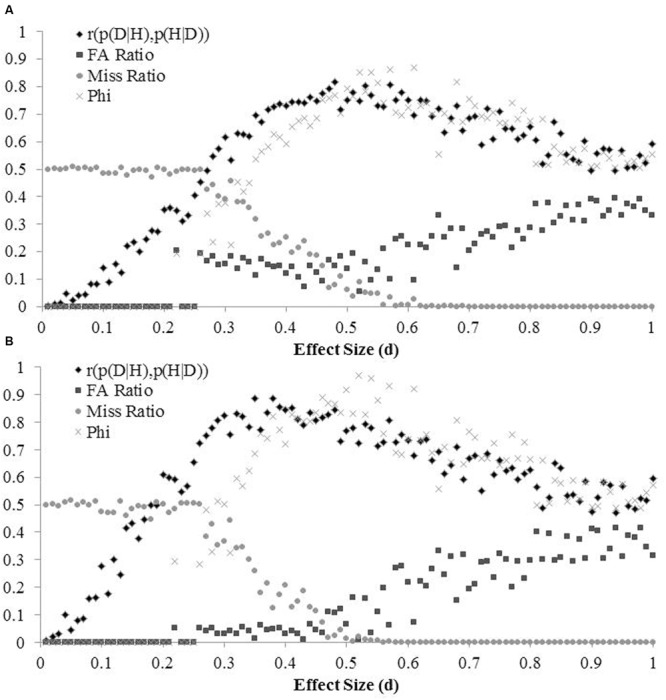
Error rates, the criterion correlation, and the accuracy correlation (phi) over 100 effect sizes (0.01 to 1 in steps of 0.01). The sampling proportion varied from 0.1 (10% of samples from H) to 0.9 (90% of samples from H) in steps of 0.1. **(A)** p(H) varied from 0.01 to 0.99 in steps of 0.01 for each effect size. **(B)** Displays the same variables after revising p(H|D) using the posterior obtained under uniform assumptions.

Both conditional probabilities of the data, p(D|H) and p(D|∼H), were independent of the prior probability of the hypothesis, p(H). The overall correlation observed between the two conditional probabilities was 0.200. Of central interest were the criterion correlations between the *p-*value and its inverse conditional, p(H|D), computed for each effect size using likelihood ratios. The mean of these correlations, after Fisher’s *r*-Z-*r* transformation, was 0.571, mean linear *R*^2^ = 0.34, mean polynomial *R*^2^ = 0.46. **Figure 3A** plots this correlation, the two error ratios (False Alarm and Miss), and the phi correlations capturing overall categorical accuracy over variations in effect size.

We then returned to the issue of risky vs. safe research in contexts where the tested hypothesis is a statistical null. Researchers often know the difference between a good bet against the null hypothesis and a long shot. To model their inferences, we departed from assuming a uniform prior distribution of p(H). Instead, we assumed that researchers had learned enough to consider a bimodal distribution of priors, seeing some hypotheses as being either likely or unlikely to be true, while seeing few hypotheses as equally likely to be true and false.^12^ We modeled their inference task by using the posterior probabilities of the hypothesis obtained after the first round of study (i.e., simulation) as the priors for the second round. We thereby obtained a revised value of p(H|D) for each of the 89,100 simulated experiments using the same diagnostic likelihood information as before. With this approach, the average criterion correlation increased to 0.634, mean linear *R*^2^= 0.40, mean polynomial *R*^2^= 0.54. **Figure 3B** shows the criterion correlations as well as the error ratios and the categorical accuracy correlation (phi) as a function of the effect size. Compared with the initial simulation, this second simulation, which granted some knowledge to the researcher, showed a clearer pattern. The criterion correlation increased earlier and more steeply as effect sizes increased and the false alarm ratio was lower for small effects.

Taken together, the two panels of **Figure 3** show that the *p-*values perform most poorly for small effects and best for medium effects. The prevalent type of error depends on the size of the effect. Small effects are easy to miss, whereas large effects are more likely to be falsely declared significant. The simulations reinforce the obvious point that small effects tend to yield higher *p-*values than large effects (*r* = -0.642, see **Table 4**). If a true effect is small and considered improbable *a priori* (p(H) > 0.5), the *p*-value may not be small enough to move p(H|D) below 0.5, thereby yielding an inferential Miss. Conversely, if a true effect is large and considered probable *a priori* (p(H) < 0.5), the *p*-value may be low enough to yield an inferential False Alarm (p(H|D) < 0.5). Significance testing is most efficient for medium effects (δ ≈ 0.5). Here, the risks of both types of error are low, and the phi coefficient between decisions based on the *p*-value (significant vs. not) and the estimated posterior probability of the null hypothesis (≤0.5 or >0.5) is high.

**Table 4:** Correlations for a simulation varying sampling proportion from 0.1 to 0.9, effect size from 0.01 to 1.0, and p(H) from 0.01 to 0.99.

	Sampling proportion	δ	p(H)	p(∼H)	p(D|H)	p(D|∼H)	p(H|D)	Updated p(H|D)
δ	0.000	–						
p(H)	0.000	0.000	–					
p(∼H)	0.000	0.000	–1.000	–				
p(D|H)	0.564	–0.642	0.000	0.000	–			
p(D|∼H)	–0.577	–0.636	0.000	0.000	0.200	–		
p(H|D)	0.713	–0.002	0.394	–0.394	*0.395*	–0.400	–	
Updated p(H|D)	0.767	0.000	0.279	–0.279	*0.435*	–0.444	0.969	–
Sample mean	–0.634	0.673	0.000	0.000	–0.800	–0.054	–0.593	–0.601

*The criterion correlations are in *italics*.*

To conclude this section, we estimated the criterion correlations for the two rounds of simulation by computing them over the entire set of 89,100 settings. In the initial round of simulations, *r* = 0.395, with a linear prediction being p(H|D) as 0.936p(D|H) + 0.353, *R*^2^ = 0.156. For *p*-values of 0.05, 0.01, and 0.001, the predicted probabilities of the null were 0.400, 0.362, and 0.354, respectively. A non-linear fit resulted in p(H|D) = -5.921p(D|H)^2^ + 3.531p(D|H) + 0.258, *R*^2^ = 0.273, yielding posterior probabilities of 0.522, 0.297, and 0.262. The false alarm ratio was lower (25.22%) than the miss ratio (30.70%), although the difference was smaller than in previous simulations. Overall classification accuracy, phi, was 0.438.

In the secondary round of simulations, when assuming an informed researcher, *r* increased to 0.435, with a linear prediction of 1.104p(D|H) + 0.328, *R*^2^ = 0.190, and predicted values of p(H|D) of 0.383, 0.339, and 0.329 for the three benchmarks of *p.* The non-linear model is -6.254p(D|H)^2^ + 3.845p(D|H) + 0.228, *R*^2^ = 0.304, with benchmark predictions of 0.518, 0.2704, and 0.232. The overall false alarm ratio dropped slightly to 0.233 and the overall miss ratio decreased slightly to 0.290. Phi increased slightly to 0.474. **Table 4** shows the correlations among these simulated variables, including both the initial (uniform assumptions) and ‘updated’ p(H|D).

In these simulations, the *p*-value predicted the posterior probability of the tested (null) hypothesis, but the associations were far from perfect. Second-order (non-linear) models improved prediction, indicating that the linear modeling underestimated the contribution of the *p-*value to inductive inference. Going beyond intuition and back-of-the-envelope analysis, these simulations show lawful patterns in the size of the criterion correlation and the types of error attached to imperfect prediction. We suspect that researchers rarely ask about the criterion correlation between *p* and the posterior of the null. Seeking objectivity, they might hesitate to estimate unknown probabilities. Judging from informal observation, we surmise that researchers worry most about missing effects when planning and conducting a study, whereas they worry most about reporting false effects after having published their own work or when reviewing their colleagues’ work.

## Are Large Samples Better Than Small Samples?

In empirical research, samples vary in size. Limited resources or lack of will can keep samples below levels recommended by power analysis. Contrariwise, some samples exceed the needs of significance testing or parameter estimation (Gigerenzer and Marewski, 2015). Yet, the received wisdom is that large samples are always better, perhaps because large samples resemble what they are intended to represent, namely the population. Larger samples deliver greater statistical power and produce fewer Misses. However, the power perspective obscures the question of false alarm ratios. Much of the critical literature suggests that increases in sample size will protect researchers from making false positive inferences. We ask if this is so.

Building on the foregoing simulations, we chose three effect sizes (δ = 0.2, 0.5, and 0.8), sampled observations, computed their means, and performed one-tailed *z*-tests on 20, 50, 100, or 200 of these means. We let the probability of the tested hypothesis, p(H), and the sampling parameter determine how many samples would be drawn from each distribution, ranging from 0.01 to 0.99 in steps of 0.01. As before, we assessed the criterion correlations between p(D|H) and p(H|D) and the *R*^2^ for both the linear and the non-linear models. To assess the performance of the *p-*value, we again report the two error ratios and the phi coefficients. As before, we proceeded in two steps. In step 1, the prior probability of the hypothesis, p(H), varied independently of the *p-*value. In step 2, we allowed some prior knowledge so that there was a positive correlation between p(H) and p(D|H). To accomplish this, we again used the posterior probability of the null obtained in round 1 as the prior in round 2.

The results are displayed in **Tables 5, 6** respectively for the first and the second round of simulations. The patterns were similar but clearer in the case of prior knowledge. Larger samples yielded lower *p*-values, and this effect was clearest when effect sizes were small. Importantly, the criterion correlations depended on both the size of the effect and the size of the sample. These correlations increased with sample size N for small effects, were fairly stable for medium effects, and *decreased* for large effects. This interactive pattern may violate intuition, but it highlights the need for caution when expecting large samples to be best. We see that when effects and samples are large, a low *p*-value is a poor predictor of the falsity of the hypothesis. The error ratios provide deeper insights. Perhaps surprisingly, false alarm ratios go up with sample size unless effects are small. Conversely, miss ratios are large for small effects and they decrease with sample size. The combined effects of the two types of error are seen in the phi coefficients. Phi generally tracks (as it has to) the criterion correlation, again showing that the *p-*value is at its diagnostic best for medium effects.

**Table 5:** Varying sample size and effect size.

δ	*N*	Mdn p	r(p(D|H),p(H|D))	*R*^2^ linear	*R*^2^ poly	FA ratio	Miss ratio	Phi
0.2	20	0.321	0.156	0.024	0.025	0.000	0.503	0.000
	50	0.239	0.340	0.116	0.118	0.192	0.496	0.088
	100	0.157	0.552	0.305	0.319	0.162	0.429	0.316
	200	0.079	0.743	0.552	0.644	0.106	0.222	0.662
								
0.5	20	0.134	0.643	0.414	0.445	0.147	0.340	0.476
	50	0.032	0.761	0.579	0.747	0.134	0.078	0.786
	100	0.006	0.651	0.424	0.650	0.261	0.000	0.691
	200	0.000	0.519	0.270	0.400	0.340	0.000	0.557
								
0.8	20	0.032	0.759	0.577	0.742	0.172	0.052	0.764
	50	0.002	0.584	0.341	0.506	0.285	0.000	0.644
	100	0.000	0.482	0.232	0.331	0.369	0.000	0.507
	200	0.000	0.374	0.140	0.203	0.420	0.000	0.404

*Round 1 – naïve investigator.*

**Table 6:** Varying sample size and effect size.

δ	*N*	Mdn p	r(p(D|H),p(H|D))	*R*^2^ linear	*R*^2^ poly	FA ratio	Miss ratio	Phi
0.2	20	0.321	0.300	0.090	0.091	0.000	0.507	0.000
	50	0.239	0.583	0.340	0.348	0.051	0.494	0.128
	100	0.157	0.785	0.617	0.655	0.035	0.403	0.433
	200	0.079	0.820	0.672	0.845	0.026	0.158	0.804
								
0.5	20	0.134	0.826	0.682	0.762	0.031	0.287	0.632
	50	0.032	0.772	0.595	0.840	0.079	0.020	0.899
	100	0.006	0.632	0.400	0.629	0.260	0.000	0.692
	200	0.000	0.507	0.257	0.382	0.344	0.000	0.554
								
0.8	20	0.032	0.767	0.588	0.817	0.132	0.009	0.846
	50	0.002	0.569	0.323	0.484	0.285	0.000	0.644
	100	0.000	0.478	0.228	0.325	0.364	0.000	0.511
	200	0.000	0.370	0.137	0.199	0.422	0.000	0.403

*Round 2 – experienced investigator.*

## Replicability

Simulations of significance testing can help estimate the probability of certain errors, but it falls to additional research to help answer the question of whether an error has actually occurred. Additional research addresses the question of replicability. Meant to answer limitations of single studies or sets of studies, replication research reproduces the some of the inferential patterns and problems at a higher level. Mindful of this analogy, we adapted our simulations to see whether the *p-*value can predict the outcome of replication research.

The issue of replicability cuts to the core of empirical science. While conceptions of replicability vary considerably, most scholars seem to agree that the replicability of empirical findings reflects the reliability of method and measurement, which in turn enables and constrains the validity of the empirical results (Asendorpf et al., 2013; Stroebe, 2016). As our investigation targets the properties of the *p-*value, we focus on the probability of re-attaining a statistically significant result once one such a result has been observed. Doing so, we limit ourselves to attempts at exact replication, that is, studies that might yield different *p*-values because of sampling variation and no other reason.

When considering the question of whether their findings might replicate, many researchers look to power analysis. Power analysis is a feature of the Neyman–Pearson theory of statistics. It is unknown in the Fisherian framework. Power analysis requires the stipulation of a second hypothesis, which is typically a non-null hypothesis or a ‘real’ difference. Assuming that this alternative hypothesis is true, that is, assuming that p(∼H) = 1, power analysis yields an estimate of the sample size needed to reject the hypothesis H with a desired probability (Cohen, 1988). Power analysis thereby shortcuts the question of *whether*, or *with what probability*, the alternative hypothesis might be true. Instead, it assumes the best possible case, namely p(∼H) = 1, i.e., p(H) = 0. It is also important to note that power analysis ignores the *p*-value of the original experiment. No matter if *p* was 0.05 or 0.00005, the researcher does the same power analysis, asking whether *p* will be at most 0.05 in the replication study. Thus, the *p*-value is not allowed to play any role in the power analysis approach to replicability. If we want to know if the *p-*value is associated with the probability of successful replication, we must modify the conventional power paradigm.

Whereas many researchers are naively optimistic that their findings will replicate, some scholars are staunchly pessimistic. Gigerenzer (in press, p. 11), for example, notes that “the chance of replicating a finding depends on many factors (e.g., [...], most of which the researcher cannot know for sure, such as whether the null or the alternative hypothesis is true.).” Our position is an intermediate one. We submit that researchers can use a two-step process to estimate the probability that a successful exact replication from the *p*-value of the original study (Krueger, 2001). Specifically, researchers can estimate the probability of re-attaining statistical significance by predicting p(∼H|D) from p(D|H) and then multiplying the result with the power index of 1 – β. They estimate p(H|D) by multiplying the observed *p*-value with a regression weight obtained from a simulated criterion correlation between p(D|H) and p(H|D) over a range of possibilities, take the complement of this estimate [i.e., p(∼H|D) = 1-p(H|D)], and multiply the result with the desired power coefficient. To illustrate this approach, consider two criterion correlations from the initial round of simulations (‘sampling probabilities’). The low estimate of the criterion correlation was 0.263, yielding the predicted values of 0.389, 0.365, and 0.360 for p(H|D) given the three benchmark values of *p.* The corresponding replication probabilities are 0.489, 0.508, and 0.512 if 1 – β = 0.8 and 0.550, 0.572, and 0.576 if 1 – β = 0.9. The more representative criterion correlation of 0.628, obtained under the assumption that researchers have some insight into the riskiness of their endeavor, suggests replication probabilities of 0.617, 0.662, and 0.672 for 1 – β = 0.8 and 0.694, 0.744, and 0.756 for 1 – β = 0.9. These probabilities increase inasmuch as researchers are knowledgeable before study (e.g., are able to predict effect sizes), have larger samples, and use non-linear models to predict the posterior probability of the null hypothesis. The data of replication studies then contribute to a cumulative updating of that probability (Moonsinghe et al., 2007).

The precision and the accuracy of these replicability estimates depend on judgment and experience (Miller, 2009). Some of the values we have reported may seem disappointing if researchers are naively optimistic regarding their chances to replicate a significant result (Stanley and Spence, 2014). This may be so because a study result is a recent, salient, and exciting stimulus that demands attention. As such stimuli generally compromise judgment under uncertainty (Dawes, 1988; Kahneman, 2011), misplaced optimism can be expected (Tversky and Kahneman, 1971; Moore and Healy, 2008). Commenting on his own approving summary of studies on social priming (Kahneman, 2011), Kahneman (2017) acknowledged he had “placed too much faith in underpowered studies.” Many researchers do (Bakker et al., 2016). Moreover, asking to find *p* < 0.05 in a replication study is a stringent criterion. Finding *p* = 0.055 after having found *p* = 0.045 does not mean that a bold substantive claim has been refuted (Gelman and Stern, 2006). More lenient criteria may be more realistic (Braver et al., 2014). For example, when there is a large disutility in missing a true effect, researchers can ask whether the effect has the same sign (Meehl, 1998) or whether the pooled data yield a *p*-value smaller than the one obtained with the first sample alone (Goh et al., 2016).

To review, our simulations showed that replicability is high inasmuch as (a) the research hypothesis is safe, (b) the *p*-value of the original study is low, and (c) the power of the replication study is high. We also saw that statistical regression constrains replicability. The probability of a successful replication falls below power estimates and below the complement of the *p*-value. This pattern is evident in the report of the Open Science Collaboration (2015). Regression is a fact to be respected rather than an artifact to be fought (Fiedler and Krueger, 2012; Fiedler and Unkelbach, 2014). Even a researcher who shies away from simulation-based assumptions can heuristically predict a successful replication with a probability of about 2/3.^13^

## Review and Discussion

Our goal was to learn how much the *p*-value reveals about the probability of the statistical hypothesis being true. We concur with Gelman (2013) that a casual inference from p(D|H) to p(H|D) has little justification. We found, however, that the two conditional probabilities are positively related. After replicating the criterion correlation of 0.38 in a baseline simulation, we found that the *p*-value and the posterior probability of the hypothesis are more closely linked under more realistic conditions. Many correlations were greater than 0.5, a value we considered necessary for an inferential cue to be useful. We also found that the probabilities of the two decision errors, False Alarms and Misses, depend on conditions other than the *p*-value itself. The size of the assumed effect and its prior probability are critical for the estimation of these errors. One intriguing result was that False Alarms pose a comparatively small problem. Consideration of sample size clarified this issue further. Unless effect sizes were small, larger samples invited more false positives. Large samples thereby *weakened* the *p*-value’s predicted value.

Broad conclusions that the *p*-value has no evidentiary value seem overstated. One version of this argument is that a *p*-value, however high, cannot corroborate the tested hypothesis. Indeed, we found that the proportion of Misses was nearly as large as the proportion of Correct Retentions (i.e., correct decisions *not* to reject the null) for most settings. Yet, it is difficult to argue that there is no difference between *p* = 0.8 or 0.08. Meehl anticipated this difficulty when asking “if we were to scrupulously refrain from saying anything like that [that the hypothesis is probably true], why would we be doing a significance test in the pragmatic context” (Meehl, 1998, p. 395).

Meehl (1978) had another significant insight. Noting that significance testing is conventionally used in its weak form, where the hypothesis H is a null hypothesis of no effect, he suggested a stronger use, where it is a non-null (or non-nil) hypothesis, ∼H, that must be nullified, an argument anticipated by Fisher (1956). None of the statistical operations change with this reversal of the conventional frame, but the conceptual shift is considerable. Now a significant result is a strike *against* the hypothesis of interest. In other words, this shift puts significance testing in the service of a Popperian, falsificationist, approach to research (see also Mayo, 1996, for an epistemological treatise).

It is instructive to consider the implications of the present simulation experiments for this falsificationist approach. The *p-*value would be positively related to p(∼H|D), large samples would militate *against* the survival of a theoretical hypothesis, and false negatives would be perceived to be the greatest threat. Meehl deplored that few psychological theories are precise enough to provide hypotheses to be submitted for the strong use of significance testing. Today the situation is much the same. It is an epistemic and theoretical issue, not a limitation of significance testing or the *p-*value.

Finally, we explored the chances that significance will be re-attained. Most researchers eventually ask whether an effect that was statistically significant in an initial study will also be significant in a repeated experiment. Some researchers know enough to cultivate a healthy skepticism and not assume that a significant result has proven their hypothesis. Clearly, a *p*-value of 0.05 does not mean that the probability of finding *p* < 0.05 again is 0.95.^14^ But what is it? Our simulations show that once the posterior probability of the hypothesis is estimated and a power level has been selected, one may be guardedly optimistic about the recovery of a significant result, absent the ethical and educational concerns over questionable research practices.

In research practice, replications are rarely treated probabilistically, and there is a risk of placing too much emphasis on the outcome of a single replication study. The success or failure of a replication study is often treated as the input for another all-or-none decision as to whether an effect is ‘real.’ Yet, the outcome of a replication study is itself no more decisive than the outcome of the original study. Each additional study makes a smaller incremental contribution to the cumulative evidence. Stopping research after one failed or one successful replication study resembles the much-criticized practice of stopping data collection when significance is obtained (Simmons et al., 2011). Stopping after one failed replication and concluding that a claim has been refuted (i.e., debunked as a false positive) is as questionable as the claim that the initial result proved the case. Our simulations show that a non-significant result is almost as likely to be a Miss (Type II error) as a Correct Retention. Treating each experiment as one data point, one may wish to preset a satisfactory number of experiments, run these experiments, and plot the effect sizes and *p*-values (or use other meta-analytic tools). Individual investigators, however, may find this strategy unrealistic. They struggle with the opportunities and limitations of small-sample statistics, and trust the scientific community to eventually integrate the available data. This strikes us a reasonable mindset.

Current discussions surrounding the replicability of psychological research results are, in part, an outgrowth of the NHST culture.^15^ Bayesians, who avoid categorical inferences about hypotheses, also avoid categorical inferences about the success or failure of a replication study. Bayesian methods model the gradual updating and refining of hypotheses, not their categorical acceptance or rejection. Likewise, parameter estimation methods are not concerned with testing and choosing, but with integrating the available evidence. Here, the weighted evidence of an original study and a follow-up provides the best window into nature. We conjecture that some of the skepticism about significance testing is motivated by the desire to overcome the replication crisis. If significance testing is replaced with “preferred methods,” the replication crisis is not solved; it is defined away.

Though finding heuristic validity in the *p*-value, we do not advocate a protocol where *p*-values shoulder the full burden of inference (Gigerenzer and Marewski, 2015). The practice of statistics is best understood as the judicious use of a toolbox (Gigerenzer, 2004; Senn, 2011). A strategy of “exploring small” as Sakaluk (2016) recommends, while “confirming big,” calls for the use of varying techniques whose strengths are best suited to the problem’s constraints. Data analysis and inference require experience and judgment (Abelson, 1995; Krantz, 1999). An eclectic and prudent perspective highlights the need for shared ethical standards. Researchers need to be open and capable to analyze their data from a variety of perspectives, using diverse tools. At the same time, they need to ensure that they do not report whichever method yields the most rewarding or desirable outcome (Simmons et al., 2011; Fiedler and Schwarz, 2016).

## The *p*-Value in a Post-Humean World

“Any rational evaluation of the significance test controversy must begin by clarifying the *aim* of inferential statistics.” With these words, Meehl (1998, p. 393, italics are his) opened a chapter in which he claimed that the problem is epistemology, not statistics (see also Mayo, 1996). We concur that any discussion of quantitative methods must be informed by reflections on the role of theory in empirical research. Theory is always broader than the available data. Yet, theoretically driven science and hypothesis evaluation depend on evidence. Evidence is limited (there can always be more), whereas theories and hypotheses refer – by design – to a broader, even unlimited, world. The appeal of significance testing is that it honors the need for an inductive leap from the known (the sampled data) to the unknown (a hidden reality). That is, significance testing is embedded in an enterprise of making inferences with statistics. Inferences from data to theory are “risky bets” (Gigerenzer, 2008, p. 20), decisions made under uncertainty. The researcher who (tentatively) rejects a hypothesis bets that this hypothesis is more likely to be false than true. A bettor does not pretend to know for sure.

We have suggested that the *p-*value is a heuristic cue allowing the researcher to estimate the value of the probability of interest, namely p(H|D). A heuristic approach to the reduction of uncertainty is useful if normative methods are not available or computationally too expensive. An alternative to the *p*-value is the Bayesian likelihood ratio, which yields a Bayes factor when multiplied with the prior odds of the hypotheses. If use of the *p-*value is a heuristic, then a full Bayesian analysis may be, according to the Bayesians, the fully rational operation. With perfect subjective confidence, Lindley (1975, p. 106) asserted that “The only good statistics is Bayesian statistics.” Setting aside the challenge of selecting a proper prior probability distribution, one may prefer likelihood ratios to *p*-values because they use information about both a hypothesis and its alternatives. Yet, when a specific alternative hypothesis is selected, the likelihood ratio adds surprisingly little – or nothing at all. Senn (2001, p. 200) noted that “the rank order correlation between *p*-values and likelihood ratio can be perfect for tests based on continuous statistics.” Consider the case in which theory predicts a large effect and the data fall between the hypothesis H and the alternative ∼H. Here, the likelihood ratio is confounded with the *p-*value. As the data drift toward ∼H, the *p-*value drops and so does the likelihood ratio. In simulation experiments, García-Pérez (2016) found perfect correlations between log-transformed *p*-values and likelihood ratios, concluding that this must be so because the latter is “only a transformation of the *p*-value, something that can be anticipated from the fact that, like the *p*-value, the Bayes factor [i.e., the likelihood ratio] is determined by the value of the *t*-statistic and the size *n* of the sample” (p. 11). We replicated this result in our own simulations.

Now consider a case in which theory predicts a small effect and the data lie beyond ∼H. Here, the *p*-value under H drops more gently than the probability of the data under ∼H. As a result, the likelihood ratio increases, providing growing relative support for a hypothesis that is becoming ever less likely. The correlation between the logged *p-*value and the likelihood ratio is perfectly negative.

The Bayesian default test also fails to provide much extra information. Wetzels et al. (2011) compared 855 empirical *p*-values with their corresponding default Bayes Factors [i.e., p(∼H|D)/p(H|D)]. The log-log correlation was negative and virtually perfect.^16^ Wetzels et al. (2011, p. 295) claimed that “the main difference between default Bayes factors and *p*-values is one of calibration; *p*-values accord more evidence against the null than do Bayes factors. Consider the *p*-values between 0.01 and 0.05, values that correspond to “positive evidence” and that usually pass the bar for publishing in academia. According to the default Bayes factor, 70% of these experimental effects convey evidence in favor of the alternative hypothesis that is only “anecdotal.” This difference in the assessment of the strength of evidence is dramatic and consequential.” What appears to be a difference in calibration is a rather a difference in words. Most researchers using significance tests consider *p*-values between 0.01 and 0.05 to be significant, whereas most Bayesians view the corresponding Bayes factors as reflecting “anecdotal evidence.” They use benchmarks and language suggested by Jeffreys (1961) that are no less heuristic than the benchmarks suggested by Fisher. If *p* < 0.01 were routinely required for significance, the calibration issue would be moot.^17^

Another alternative to significance testing is to abandon heuristic inferences about the probability of a hypothesis altogether. Instead, one may limit statistics to the calculation of descriptive indices such as effect size estimates, confidence intervals, or graphical displays (Tukey, 1977; Cumming, 2012; Stanley and Spence, 2014). These descriptive methods are useful tools in the statistical box, but they avoid making inferences about an uncertain future. We agree with the notion that computing such descriptive measures does little to change the epistemology (or: inference) drawn from a mean and its variability by undermining the researcher’s ability to make predictions (Mayo and Spanos, 2011; Perezgonzalez, 2015a). If significance testing were abandoned, the implications would go beyond bidding farewell to the *p*-value. Researchers would be nudged away from thinking in terms of theories and hypotheses. They would be limited to thinking about the data they can see. Those who believe that the future belongs to big data may welcome this view (e.g., Button et al., 2013), but many laboratory experimenters will doubt the attainment of omniscience.

We believe that there is a need for inductive thinking and statistical tools to support inductive inferences.^18^ Asking theoretical questions about latent populations enables the researcher to think about the processes that generate the data, which are then ready to be sampled (Fiedler, 2017). A rich psychological theory might describe the way in which the brain/mind produces measurable responses. It is the theorized psychological process that determines what kind of effect one may expect – if that alternative to the null hypothesis is true. For decades, the standard logic of inference has been that if the data are improbable under the null, they are probable under the substantive alternative. This logic appears to carry a grain of truth, the size of which varies.

Discontent with inductive inference is a recurring symptom of uncertainty aversion, which in turn can lead to contradictory complaints. Hearing that *p*-values are terrible and that, by the way, they are not low enough recalls the vacationer’s complaint that “The food was horrible – and the portions were so small!” The two complaints nullify each other. We are not concerned with the possibility that some individuals hold both types of belief but with the fact that the field appears to be open to both types. Likewise, it is odd to categorically call for the abandonment of significance testing on the grounds that the method invites categorical inferences. Making strict distinctions between methods that make strict distinctions and methods that do not is an instance of the former method and thus self-contradictory (and perhaps an instance of Russell’s 1902, paradox).

To be sure, contradictory critiques do not validate the method under investigation. Indeed, we confess an incoherence of our own. As we noted at the outset, we drew upon ideas from three discrete schools of statistical thought. The emphasis on exact *p*-values comes from the Fisherian school, the use of power analysis and decision errors comes from the Neyman–Pearson school, and the estimation of posterior probabilities of hypotheses comes from the Bayesian school. Gigerenzer (2004, in press) warned that the tools offered by these schools ought to not be ritually combined, but he did not proscribe any mixing of methods under all circumstances. Hence, our admission is only a partial one. We think that an integration of statistical analysis tools can be attempted and gainfully employed (see Cohen, 1994, for an eloquent example), and we regard our integration as mindful rather than ritualistic.^19^

Our main concern is with the future of statistical practice and how our results might inform it. We submit that the use of significance testing in experimental work with small to medium-sized samples may remain beneficial, especially in cases involving new questions, and assuming that researchers will consider a variety of options from the statistical toolbox. This conclusion resembles Fisher’s original advice (see also Cohen, 1990; Abelson, 1995; Wilkinson and The Task Force on Statistical Inference, 1999; Nuzzo, 2014; Sakaluk, 2016). In contrast, the eminent Bayesian Lindley (1975, p. 112) asserted that “all those methods that violate the likelihood principle” should be left to die. Later, one of us predicted that significance testing will be around because it has been around (Krueger, 2001). This prediction was an inductive one, and thus lacked logical force. But the data have supported it. Some critics of significance testing use *p-*values to support their arguments (e.g., Bakker et al., 2016; see Gigerenzer, in press, for a similar observation). We find this ironic but reassuring.

Much care is needed when it comes to a discussion of the limitations of significance testing and the traps they may set. One well-known concern is about the strict enforcement of the 0.05 threshold (which Fisher himself discouraged) and the all-or-none decision-making it begets. Bayesians lament the incoherence of significance testing, by which they mean – among other things – the intransitivity of inferences: if X is significantly greater than Z, but Y is not significantly greater than Z, it does not follow that X is greater than Y. We share these concerns, but regard them, as noted above, as a matter of education. Our principal concern belongs to the predictive validity of the *p*-value. We used a categorization scheme anchored on *p* = 0.05 to compute false alarm and miss ratios only for illustrative purposes.

Another concern is which types of hypothesis researchers select for study in the first place. Using prediction markets, Dreber et al. (2015) concluded that many researchers chase risky research hypotheses, which means that the statistical hypotheses they seek to reject are highly probable *a priori*.^20^ Even when these risky hypotheses turn out to be true, their effect sizes are likely small. This conjecture matches the finding that in most natural and cultural fields, the size of a desired reward is inversely related to its probability (Pleskac and Hertwig, 2014). In the context of statistical effects it is easier to imagine how many forces conspire to create small differences or low correlations (i.e., effects) than it is to imagine forces strong enough – and operating unopposed – to create large effects. When seeking significance under such conditions, some researchers bemoan nature’s uncooperativeness, while others invest resources to increase the size of their samples. Although this strong-effort strategy raises the probability of finding significance, our simulations suggest that it also raises the false alarm ratio.

Significance testers face a dilemma. In an idealized world, they find a significant result for a novel but risky hypothesis, replicate significance in the lab, publish in a high-impact journal, and see the results replicated by independent labs. Such is the journey of a hero who makes lasting discoveries. Alas, most researchers must accept reality and make a living by corroborating reasonably probable hypotheses. There is no shame in that.

## Author Contributions

JK and PH contributed equally to this article and author order was determined randomly. JK conducted literature review and theoretical analysis for this article, and drafted the main body of text. PH conducted the simulations and analyses, prepared the tables and figures, and drafted the results.

## Conflict of Interest Statement

The authors declare that the research was conducted in the absence of any commercial or financial relationships that could be construed as a potential conflict of interest. The reviewer TW and handling Editor declared their shared affiliation, and the handling Editor states that the process nevertheless met the standards of a fair and objective review.

## Footnotes

1 The “preferred methods” include frequentist and Bayesian methods that advocates of each school would regard as incommensurable.

2 Replications will fail because samples are too small to detect a true effect, or because they are large enough to expose the original result as a false positive.

3 A phenomenon must first be discovered before it can be shown to be invariant over contexts, that is, before it can be generalized.

4 If the population is finite with size N, a sample of size N is exhaustive and necessarily valid. A sample of N-1 is only slightly inferior, and a sample of *N* = 1 remains more informative than no sample at all (Dawes, 1989).

5 In the standard normal distribution, the correlation between the probability density [ϕ_z_(z), the height of the curve at point *z*] and the complement of the cumulative probability (the area under the curve to the right of *z*) is 0.965 when computed for 400 *z*-values ranging from 0 to 3.99. When both indices are log transformed, the correlation rises to 0.989.

6 There is no consensus among Bayesians as to whether probability or likelihood ratios are to be preferred.

7 We obtained p(H|D) as , where  and pdf refers to probability density function.

8 Note that here we refer to any hypothesis as the topic of rejection or retention.

9 The value of ST can be expressed in terms of Bayesian updating. The posterior odds against the null were 0.367, 0.575, and 0.563 respectively for *p* = 0.05, 0.01, and 0.001.

10 False alarm and miss ratios are frequentist indices. The tabulated data can submitted to Bayesian calculations with identical results (Gigerenzer and Hoffrage, 1995).

11 Simonsohn et al. (2013) reach the same conclusion with *p*-curve analysis. If p(H) = 1, p(D|H) is uniformly distributed. If p(H) = 0, the distribution becomes increasingly left-skewed (more small *p*-values) as effects become larger.

12 This bimodal distribution of p(H|D) can be seen against the Y-axis in **Figures 2A,B**.

13 Incidentally, 2/3 is the probability Laplace derived for repeating “a successful” event when the first event emerged against a background of perfect ignorance (Dawes, 1989; Gigerenzer, 2008).

14 However, Gigerenzer (in press) asserts that many researchers fail to muster even this minimal skepticism due to the learned and ritualistic nature of running a statistical test. Doing the dance of NHST as a ritual, they suffer the “crucial delusion that the *p*-value directly specifies the probability of a successful replication (1-p)” (p. 1).

15 This is one reason for why we include an investigation of replicability in the report.

16 See Figure 3 in Wetzels et al. (2011, p. 295). The authors did not compute a correlation coefficient for the plotted values.

17 Wetzels et al. (2011) assert that “this problem would not be solved by opting for a stricter significance level, such as 0.01. It is well-known that the *p*-value decreases as the sample size, n, increases. Hence, if psychologists switch to a significance level of 0.01 but inevitably increase their sample sizes to compensate for the stricter statistical threshold, then the phenomenon of anecdotal evidence will start to plague *p*-values even when these *p*-values are lower than 0.01.” This argument assumes that increasing sample size will lower the *p*-value while leaving the Bayes factor unchanged. How might this be the case if the *p-*value is needed for the computation of the Bayes factor? If some of the researchers had collected more data to lower *p*, then non-linearities should be seen Figure 3 in Wetzels et al.’s (2011). They are not, and neither are they seen in our simulations. It can be shown that raising N, *ceteris paribus*, lowers p(D|H) and p(D|∼H), but not at the same rate (unless the data fall precisely between H and ∼H). As a result, the ratio of the two also drops. To keep the ratio – and thus the Bayes factor – constant, ∼H would need to move away from the data. Moving the research hypothesis while collecting data in order to hold the Bayes factor constant hardly seems to be a recommendable intervention.

18 This itself is an inductive inference based on past experience, and therefore tautologically true.

19 The reader may wonder why we do not endorse a full-fledged Bayesian approach. Following orthodox sample statistics, we have treated the data and not the hypotheses as random variables. Bayesians do the opposite. Throughout our treatment, we have assumed competitive testing for sets of two *specific* hypotheses. By contrast, Bayesians consider hypothetical density distributions. As Lindley (1975, p. 108) declared, Bayesian statistics does not only supersede significance testing, but also makes “problems of point estimation disappear: the ‘estimate’ is the probability distribution and any single value is nothing more than a convenient partial description of this distribution.” See Koenderink (2016) for a more balanced view of the strengths and limitations of Bayesian statistics.

20 The finding that *p*-values tend to be high and successful replications improbable when null hypotheses have high prior probabilities is consistent with our simulation results.
